# Effect of birth plans integrated into childbirth preparation classes on maternal and neonatal outcomes of Iranian women: A randomized controlled trial

**DOI:** 10.3389/fgwh.2023.1120335

**Published:** 2023-04-06

**Authors:** Zaynab Mohaghegh, Mojgan Javadnoori, Mahin Najafian, Parvin Abedi, Ehsan Kazemnejad Leyli, Simin Montazeri, Shahla Bakhtiari

**Affiliations:** ^1^ Department of Midwifery, Nursing & Midwifery School, Ahvaz Jundishapur University of Medical Sciences, Ahvaz, Iran; ^2^ Department of Midwifery, Reproductive Health Promotion Research Center, School of Nursing and Midwifery, Ahvaz Jundishapur University of Medical Sciences, Ahvaz, Iran; ^3^ Department of Obstetrics and Gynecology, School of Medicine, Fertility, Infertility and Perinatology Research Center, Ahvaz Jundishapur University of Medical Sciences, Ahvaz, Iran; ^4^ Department of Midwifery, Menopause Andropause Research Centre, Ahvaz Jundishapur University of Medical Sciences, Ahvaz, Iran; ^5^ Department of Biostatistics, Guilan Road Trauma Research Center, Guilan University of Medical Sciences, Rasht, Iran; ^6^ Midwifery Department, Rosie Hospital, Cambridge University Hospitals NHS, Cambridge, United Kingdom

**Keywords:** birth plans, prenatal education, natural childbirth, maternal outcomes, neonatal outcomes

## Abstract

**Background:**

Involvement of women in the decision-making process during childbirth plays an important role in their physical and psychosocial preparation. A birth plan allows the woman to express her expectations and facilitates her participation in her own care. The present study is the first to assess the implementation of birth plans integrated into childbirth preparation classes in Tehran, Iran.

**Methods:**

This study is a randomized controlled clinical trial performed on 300 pregnant women at 32–33 weeks of gestation referring to four public health centers in Tehran, Iran. The participants were randomly allocated into intervention and control groups using block randomization method. A training session on the items of the birth plan checklist was held in the fifth session of childbirth preparation classes for the participants in the intervention group. Accordingly, a birth plan was prepared according to the requests of mothers. The birth plan was implemented after the women were admitted to the maternity ward. The primary outcomes were frequency of vaginal birth, mean duration of labor stages, and mean score of childbirth satisfaction. We used a checklist of maternal and neonatal outcomes, Mackey's childbirth satisfaction questionnaire, and a partogram form for data collection. Independent *t*-test, Mann–Whitney *U*-test, Chi-square test, Fisher's exact test, and logistic regression were used for data analysis.

**Results:**

Vaginal birth rates were significantly higher in women who had birth plans compared with those without (81.9% vs. 48.7%, *p* < 0.001). Also, the lengths of the first and the second stages of labor were significantly shorter in women having a birth plan (*p* = 0.02). Women in the birth plan group were significantly more satisfied with the process of labor and childbearing (*p* < 0.001), and started breastfeeding after birth earlier than those in the control group (*p* < 0.001).

**Conclusion:**

Having a birth plan and attending childbirth preparation classes can increase the rate of normal vaginal birth. Also, according to our results, women's participation in the decision- making process and fulfilling their preferences during birth can improve maternal and neonatal outcomes and childbirth satisfaction.

**Trial registration:** IRCT20190415043283N2. 2020-12-07.

## Introduction

Childbirth is a major life event for women so much so that it can create memories which will probably stay with them for a lifetime (1). Nowadays, most women prefer to manage their labor, use non-pharmacological intervention for labor pain, control their birth, and experience a unique birth by sharing this particular experience with their partners (2). The routine use of medical interventions such as intravenous fluids, fetal monitoring, induction or augmentation of labor, and episiotomy in hospitals have been considered as interventions that may affect women's decision-making abilities about their birth process and hence taking away their autonomy (3, 4).

Over the past decades, there has been an unprecedented and significant rise in cesarean birth rates across the world (5). Therefore, international policies have been developed to promote vaginal birth, and different approaches have been implemented to encourage this type of birth (6). Sufficient investment in childbirth preparation is key to having a birth without intervention (7). Educating pregnant women and involving them in the process of decision-making during childbirth plays an important role in their physical and psychosocial preparation (8). Both childbirth educational classes and birth plan are part of antepartum preparations which aim to provide an opportunity for education on pregnancy and offer options for labor management during childbirth (9). According to available evidence, women attending preparation courses for childbirth are better adapted to labor pain, use less medications during labor, and are less likely to need instrumental vaginal birth (10). Although evidence in Iran shows that attending educational antenatal classes has been effective in empowering pregnant women (11), and that these classes could reduce fear, anxiety and depression in primiparous women (12), the rate of cesarean section in Iran is still much higher than that in most developed countries (13). Based on a systematic review, the prevalence of cesarean birth in Iran has increased six-fold from less than 7% in the 1970s to over 48% in 2018 (14). The most important reasons for Iranian women's request for cesarean birth are fear of vaginal birth and intolerance of labor pain (15).

In addition to childbirth educational classes, birth plan is one of the philosophies advocating the de-medicalization of natural processes of labor and birth (4). Using birth plan, women are engaged in their care, and they participate in a shared decision making (SDM) process during birth (16). Accumulating evidence shows that women who are more involved in their care decisions are more informed of their options and have realistic expectations about what might happen to them. In addition, they choose options that are most valuable to them (17). Birth plan is a written document prepared by a woman during pregnancy which is a description of her expectations and preferences during childbirth (18). Birth plans generally include information such as where a woman wishes to give birth, who will attend the birth, and what forms of medical intervention and pain relief will be used (19). Deering et al., reported that the most common women requests in their birth plans were to be given the permission to walk during labor, to go through no episiotomy, to receive no pain medications or epidural, to be able to drink fluids during labor, and no continuous fetal monitoring (20).

Planning birth during the antenatal period promotes health education and fosters communication between women and health professionals (21). The World Health Organization (WHO) recommends birth plans as a part of prenatal care (22). The wide use of such planning can mitigate excessive medicalization during childbirth and empower women to be the decision-maker in their own childbirth (23). Although the use of a birth plan is commonplace in developed countries, it is rather new in developing countries (24). There are only a few studies in the world that have evaluated the effectiveness of birth plan (25, 26). Two studies in Catalonia, Spain, have reported that 86.9%–98.8% of mothers receive birth plan information from midwives during prenatal care (27, 28). In the USA and Europe, only 12%–39.8% of the women are offered a birth plan when they are admitted to hospital (29).

Despite the fact that around 90% of Iranian women give birth at hospital, unnecessary medical interventions are still very common during normal labor and childbirth, compared to developed countries (30). It is well established that when a woman is admitted in maternity ward in Iran, she is subjected to restrictive policies and has to undergo a series of routine medical procedures. For example, performing episiotomy and using oxytocin during labor without women's informed consent are common practices in Iran. In addition, according to one study, almost half of women reported that they had not even had the right to move and choose birthing positions during labor (31).

The high rate of cesarean section has raised serious concerns among health policy-makers and decision-makers in Iran (32), and the health system needs to undertake appropriate initiatives in this respect (13). Numerous attempts have been made in recent years in order to decrease the rate of unnecessary cesarean sections in Iran. These include establishing mother-friendly hospitals, setting standard protocols for labor and birth, offering preparation classes for women, midwives, and gynecologists, and holding workshops for specialists and midwives through the “Health Transformation Plan” (33). In spite of all these attempts, the rates of cesarean section are still high (14), and Iran's population is already rapidly aging (34). Previous studies on the effect of birth plan resulted in contradictory results (29, 35, 36). Hidalgo-Lopezosa et al., for example, reported that there were no significant differences between groups with and without birth plan for any of the obstetric outcomes or 5-min Apgar scores (36), while Afshar et al. found that women who attended childbirth education classes and had a birth plan, had higher odds of vaginal birth (35). Given that the use of birth plan has not been studied in Iran, involving women in the decision-making process in their labor through birth plan can be considered a strategic intervention, which may improve maternal and neonatal outcomes. This study was therefore designed to investigate the effect of birth plan along with prenatal preparation classes on maternal and neonatal outcomes. We hypothesized that using birth plan along with prenatal preparation classes can reduce the rate of unnecessary cesarean section and improve maternal and neonatal outcomes.

## Methods

### Trial design and participants

This randomized controlled trial which included two parallel groups was conducted on 300 pregnant women at gestational age of 32–33 weeks in Tehran, Iran. This study was carried out in four public health center of Tehran from December 2020 to the end of June 2021.

Eligible women to participate in the study were primiparous or multiparous women who were married and aged ≥18 years, had low-risk singleton pregnancy, were at gestational age of 32–33 weeks, had basic literacy, were planning to have normal vaginal birth, and attended the fifth session of child birth preparation classes. Women with previous cesarean section who were willing to have vaginal birth were also recruited. Women with any contraindications to vaginal birth, history of abortion, multiple pregnancy, pre-eclampsia or eclampsia, placenta previa, placental abruption, history of infertility, history of medical disorders such as cardiovascular, renal, liver, brain diseases, and abnormal fetus were excluded from the study.

### Setting

This study was conducted in four public health centers (Meysam, Ayat, Afarinesh, and Azadegan) affiliated to Tehran University of Medical Sciences. Tehran is the capital of Iran which is also known as the most populous city in this country (37). These health centers provided childbirth preparation classes and were designated for sampling in this study. Two of these centers were in south of Tehran, one in Eslamshahr, and another in Ray. In Iran, participation in childbirth preparation classes is on a voluntary basis and free, and the classes are held in eight 90 min sessions from 20 to 37 weeks of gestation. Based on women's gestational age, the following topics are covered in the classes: Anatomic and physiologic changes in pregnancy, Personal hygiene, Nutritional needs of pregnant women, Fetal growth and development, Pregnancy risk factors, Planning for childbirth, Physical and mental health, Stages and benefits of natural childbirth, Pain relief techniques, Postpartum care, and Neonatal care. Women also learn about various skills such as stretching exercises, relaxation methods, posture correction exercises, massage, and breathing techniques at the end of each session. Pregnant women are trained by skilled midwives based on standard content that is set by the Iranian Ministry of Health (38).

Two private hospitals and one hospital affiliated to the armed forces in Tehran (Omid, Ansari, and Najmiyeh) were designated for the participants’ childbirth. Although birth plan is not routinely implemented in Iran, these hospitals were chosen because they allowed pregnant women to give childbirth in maternity wards based on their birth plan. In the study period, the childbirth rates were about 2,000, 3,000, and 5,000 births annually in Omid, Ansari, and Najmiyeh hospitals, respectively. In these hospitals, obstetricians had direct responsibility for prescriptions during labor and birth. The vaginal births were carried out by midwives or obstetricians. Eligible pregnant women were selected from the above-mentioned educational classes. The first investigator (ZM, PhD student) briefed the women on the study objectives and methods, and written informed consent was obtained from those who were willing to participate. Then, a demographic questionnaire was completed for each eligible woman through interview.

### Intervention and follow-up

After assigning the participants into study groups, the researcher (ZM) held a training session for the intervention group in the fifth session of childbirth preparation classes, and all items of the birth plan checklist were explained. Training on the birth plan was held in groups, and an average of 8–10 women participated in each group. Childbirth preparation classes were held by a designated midwife, and the researcher only introduced and taught the birth plan.

Birth plans were prepared from this session up to several weeks prior to labor. In these sessions, the researcher, the pregnant women, and her husband discussed various aspects of labor and birth along with personal expectations and concerns. The specific issues that were considered and documented in each birth plan included woman's expectations and preferences (e.g., hospital selection, birth attendances, clothes, support person, and pain relief techniques), care during the first stage of labor (e.g., use of birth ball, use of pool, food, hydration, bathing and mobility during labor), care during the second stage of labor (e.g., type of pushing, position of delivery, and episiotomy), and care after delivery (e.g., baby care, first one carrying the baby, first feeding of baby and hospital discharge). Apart from predetermined items, any further needs or expectations were described in a blank space. The birth plan checklist was filled out by the women themselves. In addition, a phone number was provided to the women so that they could contact the first investigator in case they had any question. From the time the birth plan was developed until the time of birth, ZM was in contact with the participants by telephone, and she answered any question the participants had about birth plan. The women were requested to call one of the researchers (ZM) once they were admitted to the hospital for labor and birth. Upon admission to the hospital, the women brought their documented birth plans to the maternity ward and handed it to the midwife, who had already been instructed to use the plan as a basis for care. In addition, the women's birth plan was shared with an obstetrician who was responsible for the care of these women. Labor and birth management were based on the mother's requests in her birth plan.

Women in the control group received only routine care according to the hospital policy. They attended childbearing preparation classes without any birth plan. Due to the COVID-19 pandemic, some sessions of educational classes for both groups were held virtually. During the childbirth process, birth information, partogram form, and maternal and neonatal outcomes were recorded for all participants by two research assistants who were blinded to the grouping. Also, women's satisfaction with childbirth was assessed according to Mackey's Childbirth Satisfaction Rating Scale, 12–24 h after birth and before discharge from hospital. In both groups, vaginal births were attended by midwives or obstetricians in the maternity wards of the mentioned hospitals, and the researchers were not involved in providing any care during pregnancy, labor, birth, and postpartum periods.

### Outcomes

#### Primary outcomes

The primary outcomes were (1) comparison of birth mode in the studied groups (2) comparison of the mean duration of the labor stages in the studied group (3) comparison of the mean score of child birth satisfaction in the studied groups.

#### Secondary outcomes

Secondary outcomes were (1) frequency of labor augmentation, perineal tears, and maternal outcomes (2) neonatal admission to neonatal intensive care units (NICU) (3) neonatal Apgar score in the first and fifth minutes (4) initiation of breastfeeding within 1 h after birth.

### Sample size

The sample size was calculated according to the predicted reduction of cesarean section rate from 50.57% to 30% according to the instruction of the Iranian health care reform-executive (32). With the assumption of *α* = 0.05 and power = 90%, the sample size in each group was calculated to be 127. Considering 15% potential attrition rate, 150 participants were considered for each group.

### Randomization and allocation concealment

#### Sequence generation

Randomization was performed using the “blockrand” package of R software, with block sizes of four and six and an allocation ratio of 1: 1 for the intervention and control groups.

#### Allocation concealment

For allocation concealment, the type of intervention was written on a piece of paper and was placed inside consecutively numbered opaque envelopes which were kept by a person who was not aware of the objectives of the study. Therefore, neither the researchers nor the participants were aware of grouping until the commencement of the study. After informed consent was obtained from eligible women, the envelopes were opened, and the intervention started.

#### Implementation

The allocation sequence was determined by a person who was neither involved in the sampling and data collection nor aware of the study process.

#### Blinding

Due to the nature of the study, the participants and researchers could not be blinded; however, outcome assessors and data analyzer were blinded to grouping.

### Data collection tools

#### Demographic and obstetric characteristics questionnaires

The demographic questionnaire included questions about age, educational attainment, occupation, ethnicity, and body mass index. Some obstetric characteristics such as gravidity, parity, mode of pervious birth, history of prenatal care, and history of childbirth preparation classes were also recorded. The validity of this questionnaire was confirmed through content and face validity methods. The face validity was determined based on the opinions of 10 experts in the field of midwifery and reproductive health specialists. Also, the specialists reviewed the questionnaire items in terms of difficulty level, irrelevancy, and ambiguity. If necessary, the items were modified based on the given recommendations (39), and for content validity, recommendations of 10 specialists were followed in terms of grammar, using appropriate and correct words, applying correct and proper order of words in items, and appropriate scoring (40).

#### Birth plan checklist

Birth plans have different formats including a list of options that women can choose during labor and birth, and include a few open-ended questions that women may not find in the checklist (25).

Since birth plan has not been yet established in Iran, the birth plan checklist in the present study was prepared based on available evidence and clinical experience. The birth plan checklist included 13 questions. It was later reviewed by 14 experts in the field of midwifery and obstetrics, and its content validity ratio (CVR) and content validity index (CVI) were assessed. CVI values were calculated by determining the simplicity, relevance, and clarity of the items, and were scored according to a four-point Likert scale. A CVI value higher than 0.79 was considered acceptable. To determine CVR scores, the specialists were asked to comment on the necessity of each item based on a three-point Likert scale. Based on the Lawshe Table (41), the minimum acceptable CVR value was set to be 0.51. After validity assessment, questions with CVI and CVR values lower than the limit were corrected and modified. All questions (13 questions) had appropriate CVI and CVR, and no questions were removed. Finally, the CVI and CVR values for the birth plan checklist were calculated to be 0.96 and 0.85, respectively.

#### Partogram form

Partogram is a valuable tool used by midwives and obstetricians for recording labor details. The application of partogram in developed and developing countries led to its worldwide recognition (42). In order for the early detection of prolonged labor and prevention of any complications, consistent use of partogram by healthcare professionals is of critical importance (43). Each partogram consists of three main sections: fetal health status, maternal health status, and progress of labor (44). In our study, duration of labor stages, results of internal examination, and perineal status were recorded in the partogram.

#### The maternal and neonatal outcome checklist

The maternal and neonatal outcome checklist included information about mode of birth, reasons of cesarean section, admission to NICU, intrauterine growth retardation (IUGR), breastfeeding initiation, Apgar score, and neonate's weight, height, and head circumference. The content and face validity of this checklist was assessed. The face validity was confirmed based on the opinions of faculty members who were midwifery and reproductive health specialists. They reviewed the tool items based on the difficulty level, irrelevancy, and ambiguity criteria. If necessary, the items were modified based on the recommendations provided (39), and for content validity, the recommendations of 10 specialists (midwives or reproductive health specialists) about the following points were taken into account: grammar, using appropriate and correct words, applying correct and proper order of words in items, and appropriate scoring (40).

#### Mackey’s childbirth satisfaction rating scale

This scale which consists of 40 questions was developed to measure women's satisfaction from childbirth. A five-item Likert scale was used for scoring this scale in the present study. For questions 1–34, the responses were scored from 1 (very dissatisfied) to 5 (very satisfied).

For questions 37–40, a four-item Likert scale was used for scoring, which ranged from (1): very negative, to (4): very positive. A total score ≥12 indicated positive experiences and scores <12 indicated negative experiences (45). A total score ≥136 indicated satisfaction, while scores <136 indicated dissatisfaction. Questions 35–36 were open-ended and addressed any other experiences that women liked to express. The validity and reliability of Mackey's Childbirth Satisfaction Rating Scale were assessed by Goodman in 2003 (46). In Iran, the psychometric assessment of this questionnaire was carried out by Moudi et al., and its reliability was confirmed by obtaining a Cronbach's alpha of 0.78 (47). The birth plan checklist was added to the Supplementary Files.

### Data analysis

The data were analyzed using SPSS version 22 (SPSS Inc., Chicago, IL, United States). The Kolmogorov–Smirnov test was used for testing normality of data. The gestational age at birth, birth weight, and length of labor did not have normal distribution and were compared using the Mann–Whitney *U*-test. However, for data with normal distribution, the independent *t*-test was used. Chi-square test or Fisher's exact test was used for comparing categorical data. Logistic regression was used to estimate the effect of birth plan on gestational outcomes after adjusting for potential confounders (i.e., previous cesarean section, educational attainment, and maternal outcomes). In all analyses, *p* < 0.05 was considered statistically significant.

## Results

### Participants

**Figure 1:**
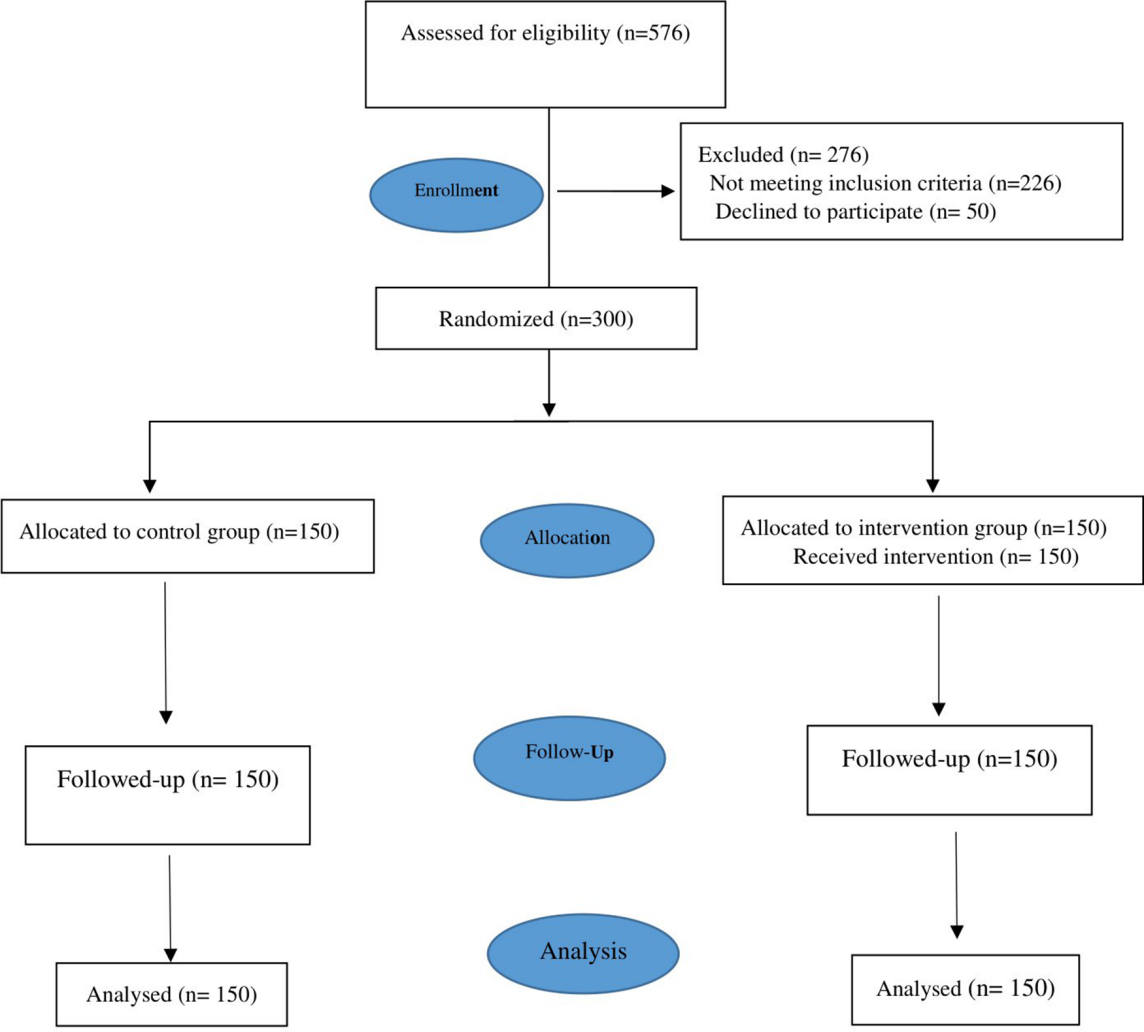
Flow-diagram of recruitment and retention of participants in the study.

Between December 2020 and June 2021, 300 eligible women were enrolled and randomly assigned to intervention (*n* = 150) or control (*n* = 150) groups (Figure 1). None of the participants dropped-out from the study, and 114 (38%), 145 (48.13%), and 41 (13.7%) of the participants were admitted to Ansari, Omid, and Najmiyeh hospitals, respectively (Table 1).

**Table 1:** Baseline demographic and obstetric characteristics of intervention and control groups.

Characteristics	Intervention group (*n* = 150)	Control group (*n* = 150)	*p*-value
	*n* (%) or mean ± SD	*n* (%) or mean ± SD	
**Age, years**	29.11 ± 4.72	28.90 ± 4.81	0.708^a^
Gestational age at birth	38.5 ± 3.13	38.7 ± 1.48	0.891^b^
Body mass index, kg/m^2^	25.12 ± 3.73	25.17 ± 4.18	0.0915^a^
**Education level**			
Primary and secondary school	2 (1.30)	17 (11.30)	
High school Diploma	28 (18.70)	36 (24.00)	<0.001^c^
University degree	120 (80.00)	97 (64.70)	
**Employment status**			
Housewife	119 (79.30)	131 (87.30)	
Employed	31 (20.70)	19 (12.70)	0.063^d^
**Economic status**			
Poor	5 (3.30)	11 (7.30)	
Moderate	119 (79.30)	122 (81.30)	0.124^d^
Good	26 (17.30)	17 (11.30)	
**Parity**			
0	100 (66.7)	84 (56.00)	
1	34 (22.7)	48 (44.00)	0.106^c^
2	12 (8.00)	13 (8.7)	
≥3	4 (2.7)	5 (3.30)	
Previous C-section	12 (8.00)	12 (8.00)	
First prenatal care (weeks)	9.19 ± 3.24; (2–27)	9.39 ± 4; (4–27)	0.646^a^
**Birth attendant**			
Obstetrician	36 (29.2)	21 (28.7)	0.94^c^
Midwife	87 (70.7)	52 (71.2)	
**Hospital**			
Ansari	64 (42.7)	50 (33.3)	
Omid	80 (53.3)	65 (43.3)	<0.001^c^
Najmiyeh	15 (10)	26 (17.3)	

Values are expressed as no (%) or mean (standard deviation); (minimum to maximum) unless otherwise stated. BMI, body mass index; C-section, cesarean section; CBE, childbirth education.

^a^ Independent *t*-test.

^b^ Mann Whitney *U* test.

^c^ Chi-square test.

^d^ Fisher's exact test.

Baseline characteristics of the participants are described in Table 1. The mean ± SD age of women in the intervention group was (29.11 ± 4.72) compared to (28.90 ± 4.81) in control group (*p* = 0.708). The majority of women in both groups were housewives (79.3% and 87.3%, respectively). Significantly more women in the birth plan group had university education compared to the control group (*p* < 0.001). The obstetric history showed that 22.7% and 32% of women in the intervention and control groups were primigravidae, respectively (*p* = 0.106).

Mean gestational week at the first prenatal care was 9w + 3d in both groups. Also, there was no difference between the two groups in terms of gestational age at birth (Table 1).

### Maternal outcomes

Table 2 shows the maternal outcomes of the participants. Vaginal birth rates were significantly higher in women who had birth plan compared to the control group (81.9% vs. 48.7%, *p* < 0.001). Also, women with a birth plan were more likely to have vaginal birth after cesarean section (VBAC) (4% vs. 1.3%, *p* < 0.001). Among those undergoing vaginal birth, the majority of women who had birth plan (70.7%) and women without birth plan (71.2%) were attended by midwives (*p* < 0.94).

**Table 2:** Maternal outcomes in intervention and control groups.

Characteristic	Intervention group (*n* = 150)	Control group (*n* = 150)	*p*-value
	*n* (%) or mean ± SD	*n* (%) or mean ± SD	
**Birth mode**			
Normal vaginal birth	123 (81.9)	73 (48.7)	
C-section	27 (18.1)	77 (51.3)	<0.001^a^
VBAC	6 (4)	2 (1.3)	
Operative vaginal delivery	2 (1.3)	1 (0.6)	
**Cervical condition At the time of admission**			
Dilatation (cm)	4.54 ± 2.14	3.94 ± 1.64	<0.063^b^
Effacement (percent)	50/06 ± 19.66	42.95 ± 17.93	<0.018^b^
Augmentation of labor	44 (33.1)	55 (45.5)	0.043^a^
**Perineal status**			
Episiotomy	55 (43)	42 (58.3)	<0.001^a^
1st degree laceration	23 (18)	5 (6.9)	<0.001^a^
2nd degree laceration	7 (5.5)	14 (19.4)	<0.001^a^
**Length of labor, minutes**			
Stage 1	218.54 ± 156.54	269.41 ± 168.83	0.02^b^
Stage 2	45.29 ± 28.53	56.18 ± 35.75	0.024^b^
Stage 3	5.33 ± 2.06	7.65 ± 5.02	<0.00^b^
Total duration	269.66 ± 164.66	338.04 ± 175.714	0.004^b^
**Pregnancy complication**			
Preeclampsia	3 (2.00)	3 (2.00)	
Rapture of the uterus	1 (0.67)	0 (0.00)	
Preterm labor	4 (2.67)	4 (2.67)	
Oligohydramnios	1 (0.67)	1 (0.67)	0.352^c^
Cord prolapse	1 (0.67)	0 (0.00)	
Hypertensive disorders	1 (0.67)	0 (0.00)	
Eclampsia	0 (0.00)	6 (4.00)	
Premature rupture of membrane	0 (0.00)	1 (0.67)	
Placenta abruption	0 (0.00)	1 (0.67)	

Values are expressed as no. (%) or mean (standard deviation). C-section, cesarean section; VBAC, vaginal birth after cesarean section. Perineal status, labor enhancement, and length of labor were calculated compared to mothers who gave vaginal birth.

^a^ Chi-square test.

^b^ Mann Whitney *U* test.

^c^ Fisher's exact test.

Women with a birth plan experienced a shorter first stage of labor and shorter childbirth stages compared to the control group (218.54 ± 156.54 vs. 269.41 ± 168.83 min, *p* = 0.02). Women with a birth plan were also less likely to have episiotomy (43% vs. 58.3%, *p* < 0.001) or receive oxytocin as an augmentation compared to the control group (33.1% vs. 45.5%, *p* = 0.043). There were no differences between the two groups in terms of other pregnancy outcomes such as postpartum hemorrhage, preeclampsia, eclampsia, or preterm labor (Table 2).

According to the results of logistic regression model in Table 3, women who had birth plan were 4.704 times more likely to have normal vaginal birth (OR = 4.704, 95% CI: 2.77–7.92).

**Table 3:** The unadjusted and adjusted odds ratios for variables related to normal vaginal birth.

Variables	Unadjusted analysis	*p*-value*	Adjusted analysis	*p*-value*
	Odds ratio (95% CI)		Odds ratio (95% CI)	
Birth Plan	4.494 (2.62–7.68)	0.000	4.704 (2.77–7.92)	0.000^a^
Previous C-section	0.790 (0.312–2.000)	0.618	-	
Education level	1.142 (0.748–1.742)	0.539	-	
Maternal outcomes	0.661 (0.303–1.44)	0.300	-	
Gravida	0.991 (0.565–1.738)	0.975	-	

CI, confidence interval.

* Back Wald method.

^a^ Adjusted for birth plan.

### Satisfaction with birth plan

Women with a birth plan had higher mean of satisfaction compared to women without a birth plan (158.83 ± 17.13 vs. 133.51 ± 24.48, *p* < 0.001), and they consistently received higher scores on all dimensions of the satisfaction scale. Specifically, women with a birth plan had a higher satisfaction with their birth experience (*p* < 0.001) (Table 4).

**Table 4:** Childbirth satisfaction and positive experience in intervention and control groups.

Variables	Intervention group (*n* = 150)	Control group (*n* = 150)	*p*-value*
	Mean ± SD		
Total satisfaction score	158.83 ± 17.13	133.51 ± 24.48	<0.001^a^
Self-satisfaction score	9.34 ± 1.07	7.84 ± 1.70	<0.001^a^
Satisfaction with midwife	42.52 ± 5.29	36.29 ± 7.59	<0.001^a^
Satisfaction with physician	37.22 ± 5.51	30.97 ± 7.71	<0.001^a^
Satisfaction with baby	9.49 ± 1.20	8.32 ± 1.59	<0.001^a^
Satisfaction with husband	9.39 ± 1.21	8.03 ± 1.67	<0.001^a^
Overall childbirth satisfaction	13.75 ± 1.62	11.57 ± 2.44	<0.001^a^
Positive experience score	12.32 ± 1.88	10.84 ± 2.62	<0.001^a^

Values are expressed as mean ± standard deviation. Total score of satisfied ranged ≥136 points and positive experience ranged ≥12.

^a^ Independent *t*-test.

* The type of test is for the values of the variables in the *p*-value column.

### Neonatal outcomes

Table 5 shows the neonatal outcomes. There were no differences between the two groups in terms of the 1st and 5th minute APGAR scores of neonates (*p* = 0.163, *p* = 0.483). Although the neonatal admission to NICU was fewer in the birth plan group (8% vs. 13.7%, in the birth plan and control groups, respectively), the difference was not significant (*p* = 0.19). Women in the intervention group started breastfeeding after birth sooner than those in the control group (*p* < 0.001).

**Table 5:** Neonatal characteristics, and outcomes in intervention and control groups.

Variables	Intervention group (*n* = 150)	Control group (*n* = 150)	*p*-value*
	Mean ± SD or *N* (%)		
**Fetal sex**			
Male	78 (52%)	80 (53%)	0.817^a^
Female	72 (48%)	70 (46.7%)	
Birth weight (g)	3,194.39 ± 390.88	3,321.35 ± 398.23	0.006^a^
Neonates’ height (cm)	50.32 ± 1.90	50.29 ± 1.80	0.508^a^
Head circumference (cm)	34.10 ± 1.33	34.40 ± 1.32	0.014^a^
1st min. Apgar	8.95 ± 0.31	8.89 ± 0.53	0.163^a^
5th min. Apgar	9.97 ± 0.24	9.94 ± 0.39	0.483^a^
Initiating breastfeeding within 1 h after birth	122 (81%)	70 (46.7%)	<0.001^a^
NICU admission	12 (0.8)	19 (12.7%)	0.274^b^
Mortality	0 (0)	1 (0.7%)	0.274^b^

Values are expressed as no. (%) or mean (standard deviation). NICU, neonatal intensive care unit.

^a^ Chi-square test.

^b^ Fisher's exact test.

* The type of test is for the values of the variables in the *p*-value column.

## Discussion

This study assessed the effect of birth plans along with childbirth preparation classes on maternal and neonatal outcomes of Iranian pregnant women. Birth plans increased the frequency of vaginal childbirth, reduced unnecessary medical interventions such as induction and episiotomy, and reduced the duration of active, second, and third phases of labor. It also improved the childbirth experiences of mothers, as well as their childbirth satisfaction. However, there was no significant difference between the two groups in terms of first- and fifth-minute Apgar scores, and frequency of admission of a newborn in NICU.

This intervention was the first of its kind to be conducted in Iran. According to the results, women with a birth plan had a significantly higher rate of vaginal birth and vaginal birth after C-section (VBAC), compared with the control group. Also, after using the adjusted model of regression, our results revealed that birth plan can increase the rate of vaginal birth (OR = 4.704, 95% CI: 2.77–7.92). These findings suggest that educating women and involving them in decision making and planning for their childbirth can increase the normal vaginal birth rate. There are, however, conflicting data in the literature on the correlation between birth preparation and mode of birth. Our findings are consistent with those of Wu et al. who reported that the rate of vaginal birth in women with a stronger preference for vaginal birth was significantly higher (48). Also, our results are in agreement with those of Afshar et al. where women with a birth plan who attended child birth preparation classes had significantly more vaginal deliveries (29). It has also been reported that having a birth plan alone is not associated with the mode of birth (35).

Our results, however, are in contrast with those of Pennell et al. who found that women with a birth plan had an increased rate of cesarean births (49). This is probably due to the differences in population and the number of participants who attended childbirth preparation classes. In Pennell et al., only 76.3% of the participants attended childbirth educational classes, and the majority of them received care from an obstetrician. Moreover, other studies have shown that there are no significant differences between the two groups in terms of cesarean rate (50).

The current study showed that having birth plan is associated with less frequent use of oxytocin for augmentation, which is in agreement with Afshar et al. (35), and Pedro Hidalgo-Lopezosa et al. (51). This finding may be explained by the fact that women with birth plan were more likely to have physiological birth (28.9% vs. 3.3%, *p* < 0.001). They had an active role in managing their labor and birth, with pervious preparation and awareness. The majority of our participants were admitted to hospital when they were in the active phase and had better dilatation and effacement compared to the women without birth plan.

We found a decreased rate of episiotomy and second-degree perineal tears, and an increased rate of first-degree perineal tears in women with birth plan compared to women in the control group. These findings are similar to other studies and reveal that having a birth plan is associated with fewer episiotomies (26, 45). A recent study also found that first degree perineal tears occurred more frequently in women who had a birth plan (72.1% vs. 25.5%, *p* < 0.001) (52). Deering et al. found no difference in episiotomy rates between women using a birth plan and those who do not (25% vs. 23%, *p* = 0.83) (50). This finding may be explained by the fact that women preparing a birth plan do more massage and perineal exercises, so they are expected to suffer from fewer lacerations and episiotomy.

Our findings demonstrated that having a birth plan may reduce the length of different stages of labor, which is not consistent with Farahat et al., who found no difference between the studied groups in terms of the length of the first and third stages of labor. They also reported that length of the second stage of labor was longer in women having a birth plan (45). The findings of the current study are similar to those of a recent study in which the length of the first stage and the total length of childbirth stages were significantly shorter in women having a birth plan (26).

A possible explanation for this finding may be that prenatal training and involving women in decision making about their values in labor and birth can reduce their fear and anxiety (11). Reducing anxiety during labor can increase the secretion of endorphins and decrease adrenaline secretion, which is a very important factor in accelerating the labor process (53). Other studies have shown that support by nurse/midwife during labor has a positive effect on maternal and fetal outcomes (54).

Childbirth is one of the most important psychological events in a mother's life. Support and communication during labor increase a “woman's childbirth satisfaction” (55). This effective communication should begin at admission and be continuously improved throughout the childbirth process. Satisfaction of childbirth reflects the mother's good feelings about childbirth, which indicates feelings of participation and control, fulfillment of needs and expectations, power, empowerment and support (25). In the present study, women with a birth plan obtained higher scores on all dimensions of the satisfaction and experience of labor and birth, which is in agreement with Farahat et al., who found that positive relationship with a midwife, excellent labor support, and being part of the decision-making process were all the components contributing to satisfaction with birth (45). However, Afshar et al. reported that women with a birth plan had no greater satisfaction compared with other women. The reason for this discrepancy may be due to the fact that they had only recruited patients with higher socio-economic status (SES) (35). Birth plans could have different associations with satisfaction depending on the socio-economic status of the participants (56). Women with higher SES are accustomed to having control and choice over their life and might feel more disappointed if the birth does not go according to the plan (57). However, women at lower levels of SES have fewer opportunities for exerting their control over their life compared with higher-SES individuals. Therefore, a woman with lower SES is expected to feel more empowered by having a birth plan (56). Planning the birth during the prenatal period can promote health education and reinforce the communication between women and health professionals (21). Some studies have suggested that using a birth plan even when women's documented preferences are not fulfilled, can improve their satisfaction (24).

Results of the present study showed that women with a birth plan are more likely to start breastfeeding earlier after birth. Furthermore, their neonates were found to be less likely to be admitted to the NICU. These findings are in agreement with those of Lundgren et al.'s study in which having a birth plan was found to have a beneficial effect on neonatal outcomes (58). These findings may be attributed to the length of labor stages since short length of labor stages can improve neonatal outcomes (59), and we found that lengths of the first and second stages of labor were shorter in women with a birth plan. Results of the present study showed that there was no difference between the 1- or 5-min APGAR scores of neonates in the birth plan and the control groups. Our results are in agreement with Afshar et al. (29) who found no difference in 1- or 5-min APGAR scores between the two groups. However, contrary to our findings, Farahat et al. reported that babies born to women with a birth plan had lower APGAR scores in the first and fifth minutes after birth (45).

The present study was performed in hospitals where more than 70% of the deliveries are performed by cesarean section. The surprising results of this study suggest that Iranian women need more midwifery services, and that by fulfilling the preferences of a pregnant woman during childbirth, the possibility of vaginal birth will increase accordingly. Childbirth education alongside a birth plan can play an effective role in empowering women. Therefore, health policymakers need to change their strategies to support women's health during childbirth. Also, government support for the implementation of women's preferences during labor and birth can be an appropriate solution against the increased cesarean section rate in Iran.

## Strengths and limitations of the study

The main limitation of this study is that it was conducted in private hospitals; therefore, the results of this study may not be generalized to all hospitals including public hospitals. However, this study has several strengths such as random sampling, and it is the first study to the best of our knowledge to investigate the effects of birth plan integrated into childbirth preparation classes in Tehran, Iran.

## Conclusion

Birth plans along with childbirth preparation classes are an appropriate strategy for reducing the rate of cesarean section and improving women's satisfaction. Involving pregnant women in decision making about their preferences during labor can improve health outcomes and satisfaction.

## Data Availability

The raw data supporting the conclusions of this article will be made available by the authors, without undue reservation.
