# Supplementary material for: Effect of birth plans integrated into childbirth preparation classes on maternal and neonatal outcomes of Iranian women: A randomized controlled trial
Source: Front Glob Womens Health. 2023 Apr 6;4:1120335. doi: 10.3389/fgwh.2023.1120335 (PMC10117766; doi:10.3389/fgwh.2023.1120335)
Supplement: Supplementary file 1 [file Datasheet1.pdf]

My Birth plan

A birth plan is a way of communicating with the midwives and doctors who care for you in labor. It tells them about the kind of labor you would like to have, what you want to happen and what you definitely want to avoid. Be flexible – the best birth plans recognise that things don't always go to plan.

Full name: ____________________ Husband's name: ____________________

Today's date: ____________________ Estimated delivery date: ____________________

Doctor/ Midwifery's name: ____________________ Hospital name: ____________________

My delivery is planned as:

- ☐ Vaginal
- ☐ VBAC

I'd like...

- ☐ Husband: ____________________
- ☐ Mother: ____________________
- ☐ friend: ____________________
- ☐ Doula: ____________________
- ☐ Other: ____________________

...to be with me during labor and birth

During labor I'd like...

- ☐ Music played (I will provide)
- ☐ To use of birth ball
- ☐ The room as quiet as possible
- ☐ As few interruptions as possible as few
- ☐ vaginal exams only as necessary
- ☐ To use a pool in hospital for pain relief
- ☐ To wear my own clothes
- ☐ My husband to film AND/OR take pictures
- ☐ My husband to be present the entire time
- ☐ To stay hydrated with clear liquids & ice chips
- ☐ To eat and drink as approved by my doctor
- ☐ To use a pool for giving birt

I'd like to spend the first stage of labor:

- ☐ Standing up
- ☐ Lying down
- ☐ Walking around
- ☐ In the shower
- ☐ In the bathub

For pain relief I'd like to use:

- ☐ Acupressure
- ☐ Massage
- ☐ Breathing techniques
- ☐ Cold therapy
- ☐ Hot therapy
- ☐ Medication
- ☐
- ☐
- ☐
- ☐ Quran Recitation
- ☐ Aromatherapy
- ☐ Standard epidural
- ☐ Only what I request at the time
- ☐ Whatever is suggested at the time
- ☐ Noting
- ☐

During delivery I would like to:

- ☐ Squat
- ☐ Semi-recline
- ☐ Lie on my side
- ☐ Be on my hands and knees
- ☐ Stand
- ☐ Lean on my husband

As the baby is delivered, I would like to:

- ☐ Push spontaneously
- ☐ Push as directed
- ☐ Push be guided by my midwife
- ☐ Use a mirror to see the baby
- ☐ Use whatever methods my doctor deems necessary
- ☐ Help catch the baby
- ☐ Let my husband catch the baby
- ☐ Take a film by my husband

I would like an episiotomy:

- ☐ Used only after perineal massage, warm compresses and positioning
- ☐ Performed only as a last resort
- ☐ Performed as my doctor /midwife deems necessary

Immediately after delivery, I would like:

- [ ] My husband to cut the umbilical cord
- [ ] To bank the cord blood
- [ ] To see the placenta before it is discard
- [ ]
- [ ]
- [ ]
- [ ]

If a C-section is necessary, I would like

- [ ] To make sure all other options have been exhausted
- [ ]
- [ ] To stay conscious
- [ ] To see my baby come out
- [ ]
- [ ]
- [ ]
- [ ]
- [ ]
- [ ]

I would like to hold baby:

- [ ] Immediately after delivery
- [ ] After being wiped clean and swaddled
- [ ]
- [ ]
- [ ]

I would like to breastfeed:

- [ ] As soon as possible after delivery
- [ ] With the help of midwife or nurse
- [ ] during first breast feeding
- [ ] Later

I would like my family members:

(names:) ______________________________

____

- [ ] To join me and baby immediately after delivery
- [ ] To join me and baby in the room later
- [ ] Only to see baby in the nursery
- [ ] To have unlimited visiting after birth

birth plan

I'd like to feed baby:

- ☐ Only with breastmilk
- ☐ Only demand
- ☐ On schedule
- ☐

I'd like baby to stay in my room:

- ☐ All the time
- ☐ During the day
- ☐ Only when I'm awake
- ☐ Only for feeding
- ☐ Only when I request

I'd like my husband:

- ☐ To have unlimited visiting
- ☐ To sleep in my room

After birth, I'd like to stay in the hospital:

- ☐ In private room
- ☐
- ☐
- ☐
